# Supplementary material for: Molecular Heterogeneity in Early-Onset Colorectal Cancer: Pathway-Specific Insights in High-Risk Populations
Source: Cancers (Basel). 2025 Apr 15;17(8):1325. doi: 10.3390/cancers17081325 (PMC12026214; doi:10.3390/cancers17081325)
Supplement: Supplementary file 1 [file cancers-17-01325-s001.zip › cancers-3552443-supplementary.pdf]

Supplementary Materials:

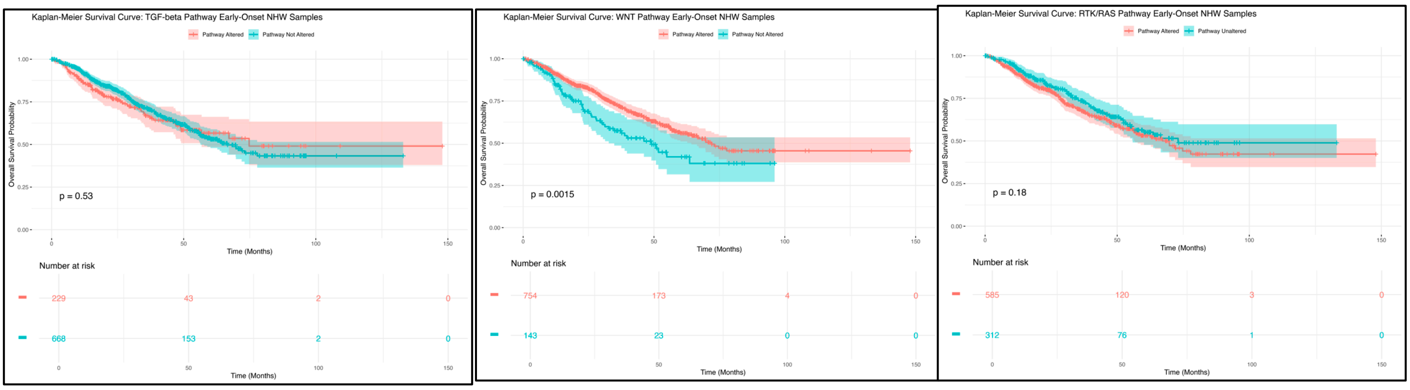

Figure S1. Overall survival trends in early-onset Non-Hispanic White (NHW) patients, categorized based on the presence or absence of alterations in the TGF-beta (left), WNT (center), and RTK/RAS (right) pathways.

Table S1. Mutation Frequencies of WNT, TGF-beta, and RTK/RAS Pathway-Associated Genes in Hispanic/Latino Colorectal Cancer Patients Stratified by early-onset colorectal cancer (EOCRC) and late-onset colorectal cancer (LOCRC).

**WNT Pathway**			
**Gene**	**Early-Onset H/L n (%)**	**Late-Onset H/L n (%)**	**p-value**
**APC Mutation**			
Present	111 (80.4%)	116 (70.7%)	0.07022
Absent	27 (19.6%)	48 (29.3%)	
**AXIN1 Mutation**			
Present	3 (2.2%)	3 (1.8%)	1
Absent	135 (97.8%)	161 (98.2%)	
**AXIN2 Mutation**			
Present	5 (3.6%)	8 (4.9%)	0.7778
Absent	131 (94.9%)	156 (95.1%)	
**GSK3B Mutation**			
Present	1 (0.7%)	1 (0.6%)	1
Absent	137 (99.3%)	163 (99.4%)	
**RNF43 Mutation**			
Present	17 (12.3%)	22 (13.4%)	0.9119
Absent	121 (87.7%)	142 (86.6%)	
**TGF-beta Pathway**			
**Gene**	**Early-Onset H/L n (%)**	**Late-Onset H/L n (%)**	**p-value**
**TGFBR2 Mutation**			
Present	11 (8.0%)	12 (7.3%)	1
Absent	127 (92.0%)	152 (92.7%)	
**TGFB2 Mutation**			
Present	0 (0.0%)	0 (0.0%)	1
Absent	138 (100.0%)	164 (100.0%)	
**TGFBRAP1 Mutation**			
Present	0 (0.0%)	0 (0.0%)	1

Absent	138 (100.0%)	164 (100.0%)	
**TGFBR1 Mutation**			
Present	4 (2.9%)	6 (3.7%)	0.7591
Absent	134 (97.1%)	158 (96.3%)	
**TGFBR3 Mutation**			
Present	0 (0.0%)	0 (0.0%)	1
Absent	138 (100.0%)	164 (100.0%)	
**TGFB1I1 Mutation**			
Present	0 (0.0%)	0 (0.0%)	1
Absent	138 (100.0%)	164 (100.0%)	
**TGFBI Mutation**			
Present	0 (0.0%)	0 (0.0%)	1
Absent	138 (100.0%)	164 (100.0%)	
**TGFB1 Mutation**			
Present	0 (0.0%)	0 (0.0%)	1
Absent	138 (100.0%)	164 (100.0%)	
**TGFBR3L Mutation**			
Present	0 (0.0%)	0 (0.0%)	1
Absent	138 (100.0%)	164 (100.0%)	
**TGFB3 Mutation**			
Present	0 (0.0%)	0 (0.0%)	1
Absent	138 (100.0%)	164 (100.0%)	
**TGFA Mutation**			
Present	0 (0.0%)	0 (0.0%)	1
Absent	138 (100.0%)	164 (100.0%)	
**SMAD1 Mutation**			
Present	0 (0.0%)	0 (0.0%)	1
Absent	138 (100.0%)	164 (100.0%)	
**SMAD2 Mutation**			
Present	7 (5.1%)	7 (4.3%)	0.955
Absent	131 (94.9%)	157 (95.7%)	
**SMAD3 Mutation**			
Present	7 (5.1%)	10 (6.1%)	0.8931
Absent	131 (94.9%)	154 (93.9%)	
**SMAD4 Mutation**			
Present	20 (14.5%)	24 (14.6%)	1
Absent	118 (85.5%)	140 (85.4%)	
**SMAD5 Mutation**			
Present	0 (0.0%)	0 (0.0%)	1
Absent	138 (100.0%)	164 (100.0%)	
**SMAD6 Mutation**			
Present	0 (0.0%)	0 (0.0%)	1
Absent	138 (100.0%)	164 (100.0%)	
**SMAD7 Mutation**			
Present	0 (0.0%)	0 (0.0%)	1
Absent	138 (100.0%)	164 (100.0%)	
**SMAD8 Mutation**			

Present	0 (0.0%)	0 (0.0%)	1
Absent	138 (100.0%)	164 (100.0%)	
**SMAD9 Mutation**			
Present	0 (0.0%)	0 (0.0%)	1
Absent	138 (100.0%)	164 (100.0%)	
**BMPR2 Mutation**			
Present	0 (0.0%)	0 (0.0%)	1
Absent	138 (100.0%)	164 (100.0%)	
**BMPER Mutation**			
Present	0 (0.0%)	0 (0.0%)	1
Absent	138 (100.0%)	164 (100.0%)	
**BMP3 Mutation**			
Present	0 (0.0%)	0 (0.0%)	1
Absent	138 (100.0%)	164 (100.0%)	
**BMP2K Mutation**			
Present	0 (0.0%)	0 (0.0%)	1
Absent	138 (100.0%)	164 (100.0%)	
**BMP1 Mutation**			
Present	0 (0.0%)	0 (0.0%)	1
Absent	138 (100.0%)	164 (100.0%)	
**BMP5 Mutation**			
Present	0 (0.0%)	0 (0.0%)	1
Absent	138 (100.0%)	164 (100.0%)	
**BMP10 Mutation**			
Present	0 (0.0%)	0 (0.0%)	1
Absent	138 (100.0%)	164 (100.0%)	
**BMPR1A Mutation**			
Present	7 (5.1%)	2 (1.2%)	0.08853
Absent	138 (100.0%)	162 (98.8%)	
**BMP15 Mutation**			
Present	0 (0.0%)	0 (0.0%)	1
Absent	138 (100.0%)	164 (100.0%)	
**BMP7 Mutation**			
Present	0 (0.0%)	0 (0.0%)	1
Absent	138 (100.0%)	164 (100.0%)	
**BMP23 Mutation**			
Present	0 (0.0%)	0 (0.0%)	1
Absent	138 (100.0%)	164 (100.0%)	
**BMP4 Mutation**			
Present	0 (0.0%)	0 (0.0%)	1
Absent	138 (100.0%)	164 (100.0%)	
**BMP6 Mutation**			
Present	0 (0.0%)	0 (0.0%)	1
Absent	138 (100.0%)	164 (100.0%)	
**BMPR1B Mutation**			
Present	1 (0.7%)	0 (0.0%)	0.457
Absent	137 (99.3%)	164 (100.0%)	
**BMP8B Mutation**			

Present	0 (0.0%)	0 (0.0%)	1
Absent	138 (100.0%)	164 (100.0%)	
**BMP8A Mutation**			
Present	0 (0.0%)	0 (0.0%)	1
Absent	138 (100.0%)	164 (100.0%)	
**RTK/RAS Pathway**			
**Gene**	**Early-Onset H/L n (%)**	**Late-Onset H/L n (%)**	**p-value**
**EGFR Mutation**			
Present	5 (3.6%)	5 (3.0%)	1
Absent	133 (96.4%)	159 (97.0%)	
**ERBB2 Mutation**			
Present	11 (8.0%)	13 (7.9%)	1
Absent	127 (92.0%)	151 (92.1%)	
**ERBB3 Mutation**			
Present	9 (6.5%)	9 (5.5%)	0.8933
Absent	129 (93.5%)	155 (94.5%)	
**ERBB4 Mutation**			
Present	12 (8.7%)	8 (4.9%)	0.2728
Absent	126 (91.3%)	156 (95.1%)	
**FGFR1 Mutation**			
Present	2 (1.4%)	4 (2.4%)	0.6914
Absent	136 (98.6%)	160 (97.6%)	
**FGFR2 Mutation**			
Present	5 (3.6%)	2 (1.2%)	0.2526
Absent	133 (96.4%)	162 (98.8%)	
**FGFR3 Mutation**			
Present	5 (3.6%)	7 (4.3%)	1
Absent	133 (96.4%)	157 (95.7%)	
**FGFR4 Mutation**			
Present	3 (2.2%)	6 (3.7%)	0.5056
Absent	135 (97.8%)	146 (89.0%)	
**KRAS Mutation**			
Present	51 (37.0%)	68 (41.5%)	0.4964
Absent	87 (63.0%)	96 (58.5%)	
**NRAS Mutation**			
Present	8 (5.8%)	9 (5.5%)	1
Absent	130 (94.2%)	155 (94.5%)	
**HRAS Mutation**			
Present	2 (1.4%)	4 (2.4%)	0.6914
Absent	136 (98.6%)	160 (97.6%)	
**BRAF Mutation**			
Present	7 (5.1%)	30 (18.3%)	**0.0009188**
Absent	131 (94.9%)	134 (81.7%)	
**MAP2K1 Mutation**			
Present	5 (3.6%)	0 (0.0%)	0.01914
Absent	133 (96.4%)	164 (100.0%)	
**MAP2K2 Mutation**			

Present	3 (2.2%)	5 (3.0%)	0.7312
Absent	135 (97.8%)	159 (97.0%)	
**MAPK1 Mutation**			
Present	2 (1.4%)	0 (0.0%)	0.208
Absent	136 (98.6%)	164 (100.0%)	
**MAPK3 Mutation**			
Present	5 (3.6%)	1 (0.6%)	0.09657
Absent	133 (96.4%)	163 (99.4%)	
**SOS1 Mutation**			
Present	2 (1.4%)	6 (3.7%)	0.2973
Absent	136 (98.6%)	158 (96.3%)	
**SHC1 Mutation**			
Present	0 (0.0%)	0 (0.0%)	1
Absent	138 (100.0%)	164 (100.0%)	
**GRB2 Mutation**			
Present	0 (0.0%)	0 (0.0%)	1
Absent	138 (100.0%)	164 (100.0%)	
**MET Mutation**			
Present	2 (1.4%)	6 (3.7%)	0.2973
Absent	136 (98.6%)	158 (96.3%)	
**PDGFRA Mutation**			
Present	8 (5.8%)	3 (1.8%)	0.1196
Absent	130 (94.2%)	161 (98.2%)	
**KIT Mutation**			
Present	5 (3.6%)	1 (0.6%)	0.09736
Absent	134 (97.1%)	163 (99.4%)	
**IGF1R Mutation**			
Present	6 (4.3%)	5 (3.0%)	0.7703
Absent	132 (95.7%)	159 (97.0%)	
**RET Mutation**			
Present	3 (2.2%)	5 (3.0%)	0.7312
Absent	135 (97.8%)	159 (97.0%)	
**ROS1 Mutation**			
Present	9 (6.5%)	8 (4.9%)	0.7138
Absent	129 (93.5%)	156 (95.1%)	
**ALK Mutation**			
Present	6 (4.3%)	9 (5.5%)	0.8506
Absent	132 (95.7%)	155 (94.5%)	
**FLT3 Mutation**			
Present	6 (4.3%)	6 (3.7%)	0.9922
Absent	132 (95.7%)	158 (96.3%)	
**NTRK1 Mutation**			
Present	5 (3.6%)	5 (3.0%)	1
Absent	133 (96.4%)	159 (97.0%)	
**NTRK2 Mutation**			
Present	4 (2.9%)	6 (3.7%)	0.7591
Absent	134 (97.1%)	158 (96.3%)	
**CBL Mutation**			

Present	8 (5.8%)	2 (1.2%)	**0.04765**
Absent	130 (94.2%)	162 (98.8%)	
**ERRFI1 Mutation**			
Present	0 (0.0%)	2 (1.2%)	0.5021
Absent	138 (100.0%)	162 (98.8%)	
**NF1 Mutation**			
Present	16 (11.6%)	6 (3.7%)	**0.01547**
Absent	122 (88.4%)	158 (96.3%)	
**RASA1 Mutation**			
Present	6 (4.3%)	10 (6.1%)	0.6757
Absent	132 (95.7%)	154 (93.9%)	
**PTPN11 Mutation**			
Present	1 (0.7%)	5 (3.0%)	0.2246
Absent	137 (99.3%)	159 (97.0%)	
**RIT1 Mutation**			
Present	3 (2.2%)	1 (0.6%)	0.3349
Absent	135 (97.8%)	163 (99.4%)	
**ARAF Mutation**			
Present	2 (1.4%)	6 (3.7%)	0.2973
Absent	136 (98.6%)	158 (96.3%)	
**RAF1 Mutation**			
Present	3 (2.2%)	3 (1.8%)	1
Absent	135 (97.8%)	161 (98.2%)	
**RAC1 Mutation**			
Present	1 (0.7%)	0 (0.0%)	0.457
Absent	137 (99.3%)	164 (100.0%)	

Table S2. Alteration frequencies of genes within the WNT, TGF-beta, and RTK/RAS pathways in early-onset colorectal cancer (EOCRC) patients, comparing Hispanic/Latino (H/L) and Non-Hispanic White (NHW) populations.

**WNT Pathway**			
**Gene**	**Early-Onset H/L n (%)**	**Early-Onset NHW n (%)**	**p-value**
**APC Mutation**			
Present	111 (80.4%)	685 (76.4%)	0.3434
Absent	27 (19.6%)	212 (23.6%)	
**AXIN1 Mutation**			
Present	3 (2.2%)	24 (2.7%)	1
Absent	135 (97.8%)	873 (97.3%)	
**AXIN2 Mutation**			
Present	5 (3.6%)	40 (4.5%)	0.8482
Absent	131 (94.9%)	857 (95.5%)	
**GSK3B Mutation**			
Present	1 (0.7%)	8 (0.9%)	1
Absent	137 (99.3%)	889 (99.1%)	
**RNF43 Mutation**			
Present	17 (12.3%)	60 (6.7%)	**0.02985**
Absent	121 (87.7%)	837 (93.3%)	

TGF-beta Pathway			
Gene	Early-Onset H/L n (%)	Early-Onset NHW n (%)	p-value
**TGFBR2 Mutation**			
Present	11 (8.0%)	38 (4.2%)	0.08765
Absent	127 (92.0%)	859 (95.8%)	
**TGFB2 Mutation**			
Present	0 (0.0%)	2 (0.2%)	1
Absent	138 (100.0%)	895 (99.8%)	
**TGFBRAP1 Mutation**			
Present	0 (0.0%)	2 (0.2%)	1
Absent	138 (100.0%)	895 (99.8%)	
**TGFBR1 Mutation**			
Present	4 (2.9%)	21 (2.3%)	0.7634
Absent	134 (97.1%)	876 (97.7%)	
**TGFBR3 Mutation**			
Present	0 (0.0%)	0 (0.0%)	1
Absent	138 (100.0%)	897 (100.0%)	
**TGFB1I1 Mutation**			
Present	0 (0.0%)	1 (0.1%)	1
Absent	138 (100.0%)	896 (99.9%)	
**TGFBI Mutation**			
Present	0 (0.0%)	0 (0.0%)	1
Absent	138 (100.0%)	897 (100.0%)	
**TGFB1 Mutation**			
Present	0 (0.0%)	0 (0.0%)	1
Absent	138 (100.0%)	897 (100.0%)	
**TGFBR3L Mutation**			
Present	0 (0.0%)	0 (0.0%)	1
Absent	138 (100.0%)	897 (100.0%)	
**TGFB3 Mutation**			
Present	0 (0.0%)	1 (0.1%)	1
Absent	138 (100.0%)	896 (99.9%)	
**TGFA Mutation**			
Present	0 (0.0%)	0 (0.0%)	1
Absent	138 (100.0%)	897 (100.0%)	
**SMAD1 Mutation**			
Present	0 (0.0%)	1 (0.1%)	1
Absent	138 (100.0%)	896 (99.9%)	
**SMAD2 Mutation**			
Present	7 (5.1%)	41 (4.6%)	0.9653
Absent	131 (94.9%)	856 (95.4%)	
**SMAD3 Mutation**			
Present	7 (5.1%)	37 (4.1%)	0.7741
Absent	131 (94.9%)	860 (95.9%)	
**SMAD4 Mutation**			
Present	20 (14.5%)	117 (13.0%)	0.7393
Absent	118 (85.5%)	780 (87.0%)	

**SMAD5 Mutation**			
Present	0 (0.0%)	1 (0.1%)	1
Absent	138 (100.0%)	896 (99.9%)	
**SMAD6 Mutation**			
Present	0 (0.0%)	0 (0.0%)	1
Absent	138 (100.0%)	897 (100.0%)	
**SMAD7 Mutation**			
Present	0 (0.0%)	0 (0.0%)	1
Absent	138 (100.0%)	897 (100.0%)	
**SMAD8 Mutation**			
Present	0 (0.0%)	0 (0.0%)	1
Absent	138 (100.0%)	897 (100.0%)	
**SMAD9 Mutation**			
Present	0 (0.0%)	1 (0.1%)	1
Absent	138 (100.0%)	896 (99.9%)	
**BMPR2 Mutation**			
Present	0 (0.0%)	2 (0.2%)	1
Absent	138 (100.0%)	895 (99.8%)	
**BMPER Mutation**			
Present	0 (0.0%)	0 (0.0%)	1
Absent	138 (100.0%)	897 (100.0%)	
**BMP3 Mutation**			
Present	0 (0.0%)	0 (0.0%)	1
Absent	138 (100.0%)	897 (100.0%)	
**BMP2K Mutation**			
Present	0 (0.0%)	0 (0.0%)	1
Absent	138 (100.0%)	897 (100.0%)	
**BMP1 Mutation**			
Present	0 (0.0%)	3 (0.3%)	1
Absent	138 (100.0%)	894 (99.7%)	
**BMP5 Mutation**			
Present	0 (0.0%)	1 (0.1%)	1
Absent	138 (100.0%)	896 (99.9%)	
**BMP10 Mutation**			
Present	0 (0.0%)	1 (0.1%)	1
Absent	138 (100.0%)	896 (99.9%)	
**BMPR1A Mutation**			
Present	7 (5.1%)	16 (1.8%)	**0.04443**
Absent	138 (100.0%)	881 (98.2%)	
**BMP15 Mutation**			
Present	0 (0.0%)	0 (0.0%)	1
Absent	138 (100.0%)	897 (100.0%)	
**BMP7 Mutation**			
Present	0 (0.0%)	1 (0.1%)	1
Absent	138 (100.0%)	896 (99.9%)	
**BMP23 Mutation**			
Present	0 (0.0%)	0 (0.0%)	1

Absent	138 (100.0%)	897 (100.0%)	
**BMP4 Mutation**			
Present	0 (0.0%)	0 (0.0%)	1
Absent	138 (100.0%)	897 (100.0%)	
**BMP6 Mutation**			
Present	0 (0.0%)	0 (0.0%)	1
Absent	138 (100.0%)	897 (100.0%)	
**BMPR1B Mutation**			
Present	1 (0.7%)	0 (0.0%)	0.1333
Absent	137 (99.3%)	897 (100.0%)	
**BMP8B Mutation**			
Present	0 (0.0%)	0 (0.0%)	1
Absent	138 (100.0%)	897 (100.0%)	
**BMP8A Mutation**			
Present	0 (0.0%)	0 (0.0%)	1
Absent	138 (100.0%)	897 (100.0%)	
**RTK/RAS Pathway**			
**Gene**	**Early-Onset H/L n (%)**	**Early-Onset NHW n (%)**	**p-value**
**EGFR Mutation**			
Present	5 (3.6%)	16 (1.8%)	0.2702
Absent	133 (96.4%)	881 (98.2%)	
**ERBB2 Mutation**			
Present	11 (8.0%)	52 (5.8%)	0.4219
Absent	127 (92.0%)	845 (94.2%)	
**ERBB3 Mutation**			
Present	9 (6.5%)	46 (5.1%)	0.6344
Absent	129 (93.5%)	851 (94.9%)	
**ERBB4 Mutation**			
Present	12 (8.7%)	49 (5.5%)	0.1912
Absent	126 (91.3%)	848 (94.5%)	
**FGFR1 Mutation**			
Present	2 (1.4%)	22 (2.5%)	0.546
Absent	136 (98.6%)	875 (97.5%)	
**FGFR2 Mutation**			
Present	5 (3.6%)	21 (2.3%)	0.546
Absent	133 (96.4%)	876 (97.7%)	
**FGFR3 Mutation**			
Present	5 (3.6%)	24 (2.7%)	0.7256
Absent	133 (96.4%)	873 (97.3%)	
**FGFR4 Mutation**			
Present	3 (2.2%)	17 (1.9%)	0.7415
Absent	135 (97.8%)	880 (98.1%)	
**KRAS Mutation**			
Present	51 (37.0%)	371 (41.4%)	0.3751
Absent	87 (63.0%)	526 (58.6%)	
**NRAS Mutation**			
Present	8 (5.8%)	25 (2.8%)	0.1067

Absent	130 (94.2%)	872 (97.2%)	
**HRAS Mutation**			
Present	2 (1.4%)	8 (0.9%)	0.631
Absent	136 (98.6%)	889 (99.1%)	
**BRAF Mutation**			
Present	7 (5.1%)	67 (7.5%)	0.401
Absent	131 (94.9%)	830 (92.5%)	
**MAP2K1 Mutation**			
Present	5 (3.6%)	16 (1.8%)	0.2702
Absent	133 (96.4%)	881 (98.2%)	
**MAP2K2 Mutation**			
Present	3 (2.2%)	6 (0.7%)	0.1063
Absent	135 (97.8%)	891 (99.3%)	
**MAPK1 Mutation**			
Present	2 (1.4%)	7 (0.8%)	0.3423
Absent	136 (98.6%)	890 (99.2%)	
**MAPK3 Mutation**			
Present	5 (3.6%)	6 (0.7%)	**0.006833**
Absent	133 (96.4%)	891 (99.3%)	
**SOS1 Mutation**			
Present	2 (1.4%)	28 (3.1%)	0.4136
Absent	136 (98.6%)	869 (96.9%)	
**SHC1 Mutation**			
Present	0 (0.0%)	2 (0.2%)	1
Absent	138 (100.0%)	895 (99.8%)	
**GRB2 Mutation**			
Present	0 (0.0%)	0 (0.0%)	1
Absent	138 (100.0%)	897 (100.0%)	
**MET Mutation**			
Present	2 (1.4%)	19 (2.1%)	1
Absent	136 (98.6%)	878 (97.9%)	
**PDGFRA Mutation**			
Present	8 (5.8%)	36 (4.0%)	0.4591
Absent	130 (94.2%)	861 (96.0%)	
**KIT Mutation**			
Present	5 (3.6%)	30 (3.3%)	1
Absent	134 (97.1%)	867 (96.7%)	
**IGF1R Mutation**			
Present	6 (4.3%)	32 (3.6%)	0.8331
Absent	132 (95.7%)	865 (96.4%)	
**RET Mutation**			
Present	3 (2.2%)	29 (3.2%)	0.7902
Absent	135 (97.8%)	868 (96.8%)	
**ROS1 Mutation**			
Present	9 (6.5%)	39 (4.3%)	0.3612
Absent	129 (93.5%)	858 (95.7%)	
**ALK Mutation**			
Present	6 (4.3%)	50 (5.6%)	0.696

Absent	132 (95.7%)	847 (94.4%)	
**FLT3 Mutation**			
Present	6 (4.3%)	16 (1.8%)	0.1037
Absent	132 (95.7%)	881 (98.2%)	
**NTRK1 Mutation**			
Present	5 (3.6%)	22 (2.5%)	0.6056
Absent	133 (96.4%)	875 (97.5%)	
**NTRK2 Mutation**			
Present	4 (2.9%)	19 (2.1%)	0.534
Absent	134 (97.1%)	878 (97.9%)	
**CBL Mutation**			
Present	8 (5.8%)	13 (1.4%)	**0.002302**
Absent	130 (94.2%)	884 (98.6%)	
**ERRFI1 Mutation**			
Present	0 (0.0%)	12 (1.3%)	0.3854
Absent	138 (100.0%)	885 (98.7%)	
**NF1 Mutation**			
Present	16 (11.6%)	55 (6.1%)	**0.02907**
Absent	122 (88.4%)	842 (93.9%)	
**RASA1 Mutation**			
Present	6 (4.3%)	34 (3.8%)	0.937
Absent	132 (95.7%)	863 (96.2%)	
**PTPN11 Mutation**			
Present	1 (0.7%)	12 (1.3%)	1
Absent	137 (99.3%)	885 (98.7%)	
**RIT1 Mutation**			
Present	3 (2.2%)	9 (1.0%)	0.2076
Absent	135 (97.8%)	888 (99.0%)	
**ARAF Mutation**			
Present	2 (1.4%)	15 (1.7%)	1
Absent	136 (98.6%)	882 (98.3%)	
**RAF1 Mutation**			
Present	3 (2.2%)	24 (2.7%)	1
Absent	135 (97.8%)	873 (97.3%)	
**RAC1 Mutation**			
Present	1 (0.7%)	8 (0.9%)	1
Absent	137 (99.3%)	889 (99.1%)	

Table S3. Nature of gene mutations within the WNT, TGF-β, and RTK/RAS pathways in early-onset colorectal cancer (EOCRC) and late-onset colorectal cancer (LOCRC) among Hispanic/Latino (H/L) and non-Hispanic White (NHW) patients. Mutation types include frame shift deletions, frame shift insertions, missense mutations, nonsense mutations, splice site mutations, and translation start site mutations.

WNT Pathway

	Early-Onset Hispanic/Latino Samples					
	Frame Shift Deletion	Frame Shift Insertion	In Frame Del	Missense Mutation	Nonsense Mutation	Splice Site
AXIN1	0.0%	0.0%	0.0%	100.0%	0.0%	0.0%
GSK3B	0.0%	0.0%	0.0%	100.0%	0.0%	0.0%
RNF43	48.0%	0.0%	0.0%	36.0%	16.0%	0.0%
APC	23.6%	5.9%	0.5%	17.3%	51.4%	1.4%
AXIN2	16.7%	16.7%	0.0%	33.3%	16.7%	16.7%

	Late-Onset Hispanic/Latino Samples					
	Frame Shift Deletion	Frame Shift Insertion	Missense Mutation	Nonsense Mutation	Splice Region	Splice Site
AXIN1	50.0%	0.0%	50.0%	0.0%	0.0%	0.0%
GSK3B	0.0%	0.0%	100.0%	0.0%	0.0%	0.0%
RNF43	51.3%	10.3%	28.2%	10.3%	0.0%	0.0%
APC	25.3%	6.7%	2.8%	60.7%	0.6%	3.9%
AXIN2	54.5%	9.1%	27.3%	9.1%	0.0%	0.0%

	Early-Onset NHW Samples							
	Frame Shift Deletion	Frame Shift Insertion	In Frame Del	In Frame Ins	Missense Mutation	Nonsense Mutation	Splice Site	Translation Start Site
AXIN1	3.6%	0.0%	0.0%	0.0%	85.7%	7.1%	3.6%	0.0%
GSK3B	0.0%	11.1%	0.0%	0.0%	66.7%	22.2%	0.0%	0.0%
RNF43	56.3%	8.8%	0.0%	1.3%	20.0%	8.8%	3.8%	1.3%
APC	29.7%	9.0%	0.0%	0.0%	7.8%	51.3%	2.2%	0.0%
AXIN2	36.7%	16.3%	2.0%	2.0%	32.7%	6.1%	4.1%	0.0%

	Late-Onset NHW Samples								
	Frame Shift Deletion	Frame Shift Insertion	In Frame Del	In Frame Ins	Missense Mutation	Nonsens e Mutation	Splice Regio n	Splice Site	Translation Start Site
AXIN1	24.3%	5.6%	0.0%	0.0%	62.6%	3.7%	0.0%	3.7%	0.0%
GSK3B	0.0%	0.0%	0.0%	0.0%	75.8%	18.2%	0.0%	6.1%	0.0%

RNF43	59.4%	11.2%	0.5%	0.0%	16.8%	9.9%	0.0%	1.8%	0.5%
APC	26.7%	10.7%	0.1%	0.0%	5.6%	54.8%	0.1%	1.9%	0.0%
AXIN2	28.4%	20.8%	2.2%	3.3%	39.3%	1.6%	0.5%	3.3%	0.5%

TGF-beta Pathway

	Early-Onset Hispanic/Latino Samples					
	Frame Shift Deletion	Frame Shift Insertion	Missense Mutation	Nonsense Mutation	Splice Site	Translation Start Site
TGFBR1	0.0%	0.0%	85.7%	0.0%	0.0%	14.3%
TGFBR2	41.7%	0.0%	58.3%	0.0%	0.0%	0.0%
SMAD2	0.0%	0.0%	70.0%	30.0%	0.0%	0.0%
SMAD3	0.0%	0.0%	87.5%	12.5%	0.0%	0.0%
SMAD4	9.1%	9.1%	68.2%	13.6%	0.0%	0.0%
BMPR1A	0.0%	0.0%	85.7%	0.0%	14.3%	0.0%
BMPR1B	0.0%	0.0%	100.0%	0.0%	0.0%	0.0%

	Late-Onset Hispanic/Latino Samples					
	Frame Shift Deletion	Frame Shift Insertion	In Frame Del	Missense Mutation	Nonsense Mutation	Splice Site
TGFBR1	16.7%	0.0%	16.7%	66.7%	0.0%	0.0%
TGFBR2	46.2%	0.0%	0.0%	53.8%	0.0%	0.0%
SMAD2	25.0%	0.0%	0.0%	75.0%	0.0%	0.0%
SMAD3	0.0%	9.1%	0.0%	72.7%	18.2%	0.0%
SMAD4	18.5%	0.0%	0.0%	74.1%	3.7%	3.7%
BMPR1A	0.0%	0.0%	0.0%	33.3%	66.7%	0.0%

	Early-Onset NHW Samples								
	Frame Shift Deletion	Frame Shift Insertion	In Frame Del	In Frame Ins	Missense Mutation	Nonsense Mutation	Nonstop Mutation	Splice Site	Translation Start Site
TGFBRAP1	0.0%	0.0%	0.0%	0.0%	100.0%	0.0%	0.0%	0.0%	0.0%
TGFB2	0.0%	0.0%	0.0%	0.0%	66.7%	33.3%	0.0%	0.0%	0.0%
TGFBR1	3.8%	0.0%	0.0%	0.0%	65.4%	26.9%	0.0%	3.8%	0.0%
TGFBR2	33.3%	2.4%	7.1%	2.4%	52.4%	0.0%	0.0%	0.0%	2.4%
TGFB1I1	0.0%	0.0%	0.0%	0.0%	100.0%	0.0%	0.0%	0.0%	0.0%
TGFB3	100.0%	0.0%	0.0%	0.0%	0.0%	0.0%	0.0%	0.0%	0.0%
SMAD1	0.0%	0.0%	0.0%	0.0%	100.0%	0.0%	0.0%	0.0%	0.0%

SMAD2	7.7%	7.7%	0.0%	0.0%	55.8%	23.1%	3.8%	1.9%	0.0%
SMAD3	8.7%	4.3%	0.0%	0.0%	69.6%	17.4%	0.0%	0.0%	0.0%
SMAD4	3.9%	3.1%	2.3%	0.8%	75.8%	11.7%	0.0%	2.3%	0.0%
SMAD5	0.0%	0.0%	0.0%	0.0%	100.0%	0.0%	0.0%	0.0%	0.0%
SMAD9	0.0%	0.0%	0.0%	0.0%	100.0%	0.0%	0.0%	0.0%	0.0%
BMPR2	66.7%	0.0%	0.0%	0.0%	33.3%	0.0%	0.0%	0.0%	0.0%
BMP1	33.3%	0.0%	0.0%	0.0%	66.7%	0.0%	0.0%	0.0%	0.0%
BMP5	0.0%	0.0%	0.0%	0.0%	100.0%	0.0%	0.0%	0.0%	0.0%
BMP10	0.0%	0.0%	0.0%	0.0%	100.0%	0.0%	0.0%	0.0%	0.0%
BMPR1A	0.0%	5.9%	0.0%	0.0%	52.9%	35.3%	0.0%	5.9%	0.0%
BMP7	0.0%	0.0%	0.0%	0.0%	100.0%	0.0%	0.0%	0.0%	0.0%

	Late-Onset NHW Samples									
	Frame Shift Deletion	Frame Shift Insertion	In Frame Del	In Frame Ins	Missense Mutation	Nonsense Mutation	Nonstop Mutation	Splice Region	Splice Site	Translation Start Site
TGFBRAP1	14.3%	0.0%	0.0%	0.0%	85.7%	0.0%	0.0%	0.0%	0.0%	0.0%
TGFBR3	0.0%	0.0%	0.0%	0.0%	100.0%	0.0%	0.0%	0.0%	0.0%	0.0%
TGFB2	0.0%	0.0%	0.0%	0.0%	100.0%	0.0%	0.0%	0.0%	0.0%	0.0%
TGFBR1	3.6%	0.0%	7.1%	0.0%	82.1%	7.1%	0.0%	0.0%	0.0%	0.0%
TGFBR2	37.7%	2.6%	4.0%	0.0%	45.7%	6.0%	0.0%	0.0%	4.0%	0.0%
TGFB1I1	0.0%	0.0%	0.0%	0.0%	100.0%	0.0%	0.0%	0.0%	0.0%	0.0%
TGFBI	0.0%	0.0%	0.0%	0.0%	100.0%	0.0%	0.0%	0.0%	0.0%	0.0%
TGFB1	0.0%	0.0%	0.0%	0.0%	75.0%	25.0%	0.0%	0.0%	0.0%	0.0%
TGFB3	0.0%	0.0%	0.0%	0.0%	100.0%	0.0%	0.0%	0.0%	0.0%	0.0%
TGFA	0.0%	0.0%	0.0%	0.0%	100.0%	0.0%	0.0%	0.0%	0.0%	0.0%
SMAD1	25.0%	0.0%	0.0%	0.0%	75.0%	0.0%	0.0%	0.0%	0.0%	0.0%
SMAD2	9.9%	5.4%	0.0%	0.0%	55.9%	26.1%	1.8%	0.0%	0.9%	0.0%
SMAD3	4.9%	7.8%	1.0%	0.0%	69.6%	12.7%	1.0%	0.0%	2.9%	0.0%
SMAD4	10.0%	4.2%	1.1%	0.6%	68.8%	10.6%	0.3%	0.6%	3.6%	0.3%
SMAD5	0.0%	0.0%	0.0%	0.0%	100.0%	0.0%	0.0%	0.0%	0.0%	0.0%
SMAD6	0.0%	0.0%	0.0%	0.0%	100.0%	0.0%	0.0%	0.0%	0.0%	0.0%
SMAD7	50.0%	0.0%	0.0%	0.0%	50.0%	0.0%	0.0%	0.0%	0.0%	0.0%
SMAD9	0.0%	0.0%	0.0%	0.0%	100.0%	0.0%	0.0%	0.0%	0.0%	0.0%
BMPR2	44.4%	0.0%	0.0%	0.0%	27.8%	25.0%	0.0%	0.0%	2.8%	0.0%
BMPER	14.3%	0.0%	0.0%	0.0%	71.4%	0.0%	0.0%	0.0%	14.3%	0.0%
BMP3	0.0%	0.0%	0.0%	0.0%	28.6%	14.3%	0.0%	0.0%	0.0%	0.0%
BMP2K	0.0%	0.0%	0.0%	0.0%	100.0%	0.0%	0.0%	0.0%	0.0%	0.0%

BMP1	22.2%	0.0%	0.0%	0.0%	77.8%	0.0%	0.0%	0.0%	0.0%	0.0%
BMP5	0.0%	0.0%	0.0%	0.0%	66.7%	33.3%	0.0%	0.0%	0.0%	0.0%
BMP10	50.0%	0.0%	0.0%	0.0%	50.0%	0.0%	0.0%	0.0%	0.0%	0.0%
BMPR1A	11.1%	11.1%	3.7%	0.0%	48.1%	20.4%	0.0%	0.0%	3.7%	1.9%
BMP15	0.0%	0.0%	0.0%	0.0%	75.0%	25.0%	0.0%	0.0%	0.0%	0.0%
BMP7	0.0%	0.0%	0.0%	0.0%	100.0%	0.0%	0.0%	0.0%	0.0%	0.0%
BMP4	0.0%	0.0%	0.0%	0.0%	100.0%	0.0%	0.0%	0.0%	0.0%	0.0%
BMP6	0.0%	0.0%	0.0%	0.0%	40.0%	0.0%	0.0%	0.0%	0.0%	0.0%
BMPR1B	20.0%	0.0%	0.0%	0.0%	60.0%	0.0%	0.0%	0.0%	20.0%	0.0%
BMP8A	0.0%	0.0%	0.0%	0.0%	100.0%	0.0%	0.0%	0.0%	0.0%	0.0%

	Early-Onset Hispanic/Latino Samples				
	Frame Shift Deletion	Frame Shift Insertion	Missense Mutation	Nonsense Mutation	Splice Site
IGF1R	0.0%	0.0%	83.3%	0.0%	16.7%
ERBB2	6.7%	0.0%	93.3%	0.0%	0.0%
EGFR	20.0%	0.0%	60.0%	0.0%	20.0%
ERBB3	0.0%	0.0%	90.9%	0.0%	9.1%
ERBB4	4.5%	18.2%	77.3%	0.0%	0.0%
BRAF	0.0%	0.0%	100.0%	0.0%	0.0%
FGFR1	0.0%	0.0%	100.0%	0.0%	0.0%
FGFR2	0.0%	0.0%	77.8%	22.2%	0.0%
FGFR3	0.0%	0.0%	100.0%	0.0%	0.0%
FGFR4	33.3%	0.0%	50.0%	0.0%	16.7%
HRAS	0.0%	0.0%	100.0%	0.0%	0.0%
KRAS	0.0%	0.0%	100.0%	0.0%	0.0%
MAP2K1	0.0%	0.0%	100.0%	0.0%	0.0%
MAP2K2	0.0%	0.0%	100.0%	0.0%	0.0%
MAPK1	0.0%	0.0%	100.0%	0.0%	0.0%
MAPK3	0.0%	0.0%	100.0%	0.0%	0.0%
NF1	21.2%	12.1%	48.5%	9.1%	9.1%
NRAS	0.0%	0.0%	100.0%	0.0%	0.0%
NTRK1	0.0%	0.0%	100.0%	0.0%	0.0%
NTRK2	0.0%	0.0%	100.0%	0.0%	0.0%
PDGFRA	0.0%	0.0%	92.9%	0.0%	7.1%
RAC1	0.0%	0.0%	100.0%	0.0%	0.0%
RAF1	0.0%	0.0%	100.0%	0.0%	0.0%
RASA1	50.0%	0.0%	37.5%	12.5%	0.0%
SOS1	0.0%	0.0%	50.0%	50.0%	0.0%

ALK	0.0%	8.3%	91.7%	0.0%	0.0%
ARAF	50.0%	0.0%	50.0%	0.0%	0.0%
CBL	0.0%	0.0%	87.5%	0.0%	12.5%
FLT3	0.0%	0.0%	87.5%	0.0%	12.5%
KIT	0.0%	0.0%	80.0%	0.0%	20.0%
MET	0.0%	0.0%	66.7%	0.0%	33.3%
PTPN11	0.0%	0.0%	100.0%	0.0%	0.0%
RET	0.0%	0.0%	50.0%	25.0%	25.0%
RIT1	0.0%	0.0%	100.0%	0.0%	0.0%
ROS1	4.5%	0.0%	77.3%	18.2%	0.0%

RTK/RAS Pathway

	Late-Onset Hispanic/Latino Samples					
	Frame Shift Deletion	Frame Shift Insertion	In Frame Del	Missense Mutation	Nonsense Mutation	Splice Site
IGF1R	40.0%	0.0%	0.0%	60.0%	0.0%	0.0%
ERBB2	13.3%	0.0%	0.0%	86.7%	0.0%	0.0%
EGFR	0.0%	0.0%	0.0%	100.0%	0.0%	0.0%
ERBB3	0.0%	9.1%	0.0%	90.9%	0.0%	0.0%
ERBB4	10.0%	10.0%	0.0%	80.0%	0.0%	0.0%
BRAF	0.0%	0.0%	0.0%	100.0%	0.0%	0.0%
FGFR1	0.0%	0.0%	0.0%	100.0%	0.0%	0.0%
FGFR2	0.0%	0.0%	0.0%	100.0%	0.0%	0.0%
FGFR3	0.0%	28.6%	0.0%	71.4%	0.0%	0.0%
FGFR4	0.0%	0.0%	0.0%	100.0%	0.0%	0.0%
HRAS	0.0%	0.0%	0.0%	100.0%	0.0%	0.0%
KRAS	0.0%	0.0%	0.0%	100.0%	0.0%	0.0%
MAP2K2	0.0%	0.0%	0.0%	100.0%	0.0%	0.0%
MAPK3	0.0%	0.0%	0.0%	100.0%	0.0%	0.0%
NF1	25.0%	0.0%	0.0%	37.5%	0.0%	37.5%
NRAS	0.0%	0.0%	0.0%	100.0%	0.0%	0.0%
NTRK1	33.3%	0.0%	0.0%	66.7%	0.0%	0.0%
NTRK2	0.0%	0.0%	0.0%	100.0%	0.0%	0.0%
PDGFRA	0.0%	0.0%	0.0%	66.7%	33.3%	0.0%
RAF1	33.3%	0.0%	0.0%	66.7%	0.0%	0.0%
RASA1	27.3%	18.2%	9.1%	27.3%	0.0%	18.2%
SOS1	0.0%	0.0%	0.0%	100.0%	0.0%	0.0%
ALK	22.2%	0.0%	0.0%	77.8%	0.0%	0.0%
ARAF	66.7%	0.0%	0.0%	33.3%	0.0%	0.0%
CBL	0.0%	0.0%	0.0%	100.0%	0.0%	0.0%

ERRFI1	50.0%	0.0%	0.0%	50.0%	0.0%	0.0%
FLT3	0.0%	33.3%	0.0%	33.3%	33.3%	0.0%
KIT	0.0%	0.0%	0.0%	100.0%	0.0%	0.0%
MET	28.6%	0.0%	0.0%	71.4%	0.0%	0.0%
PTPN11	16.7%	0.0%	0.0%	66.7%	16.7%	0.0%
RET	0.0%	0.0%	0.0%	80.0%	0.0%	20.0%
RIT1	0.0%	0.0%	0.0%	100.0%	0.0%	0.0%
ROS1	0.0%	0.0%	0.0%	75.0%	12.5%	12.5%

	Early-Onset NHW Samples							
	Frame Shift Deletion	Frame Shift Insertion	In Frame Del	In Frame Ins	Missense Mutation	Nonsense Mutation	Splice Site	Translation Start Site
IGF1R	2.6%	2.6%	0.0%	0.0%	74.4%	17.9%	2.6%	0.0%
ERBB2	4.3%	2.2%	2.2%	0.0%	91.3%	0.0%	0.0%	0.0%
EGFR	0.0%	0.0%	0.0%	0.0%	100.0%	0.0%	0.0%	0.0%
ERBB3	6.9%	0.0%	0.0%	0.0%	89.7%	1.7%	0.0%	1.7%
ERBB4	1.4%	2.9%	0.0%	0.0%	78.6%	12.9%	4.3%	0.0%
BRAF	2.8%	2.8%	1.4%	0.0%	87.3%	2.8%	2.8%	0.0%
FGFR1	4.3%	0.0%	0.0%	0.0%	91.3%	0.0%	4.3%	0.0%
FGFR2	4.3%	0.0%	0.0%	0.0%	78.3%	17.4%	0.0%	0.0%
FGFR3	7.7%	7.7%	0.0%	0.0%	80.8%	3.8%	0.0%	0.0%
FGFR4	5.9%	0.0%	0.0%	0.0%	94.1%	0.0%	0.0%	0.0%
HRAS	0.0%	0.0%	0.0%	0.0%	100.0%	0.0%	0.0%	0.0%
KRAS	0.0%	0.0%	0.0%	0.0%	99.7%	0.3%	0.0%	0.0%
MAP2K1	6.3%	0.0%	0.0%	0.0%	93.8%	0.0%	0.0%	0.0%
MAP2K2	0.0%	16.7%	0.0%	0.0%	83.3%	0.0%	0.0%	0.0%
MAPK1	0.0%	0.0%	14.3%	0.0%	85.7%	0.0%	0.0%	0.0%
MAPK3	0.0%	0.0%	0.0%	0.0%	85.7%	14.3%	0.0%	0.0%
NF1	7.9%	13.2%	0.0%	0.0%	60.5%	13.2%	5.3%	0.0%
NRAS	0.0%	0.0%	0.0%	0.0%	100.0%	0.0%	0.0%	0.0%
SHC1	0.0%	0.0%	0.0%	0.0%	100.0%	0.0%	0.0%	0.0%
NTRK1	4.2%	4.2%	0.0%	0.0%	91.7%	0.0%	0.0%	0.0%
NTRK2	0.0%	5.0%	0.0%	0.0%	90.0%	5.0%	0.0%	0.0%
PDGFRA	2.0%	0.0%	0.0%	0.0%	81.6%	10.2%	6.1%	0.0%
RAC1	12.5%	0.0%	0.0%	0.0%	87.5%	0.0%	0.0%	0.0%
RAF1	0.0%	0.0%	0.0%	0.0%	91.7%	8.3%	0.0%	0.0%
RASA1	10.6%	2.1%	0.0%	0.0%	68.1%	14.9%	4.3%	0.0%
SOS1	8.6%	2.9%	0.0%	0.0%	74.3%	14.3%	0.0%	0.0%
ALK	11.3%	0.0%	0.0%	0.0%	78.9%	9.9%	0.0%	0.0%
ARAF	37.5%	0.0%	0.0%	0.0%	62.5%	0.0%	0.0%	0.0%

CBL	0.0%	6.7%	0.0%	13.3%	66.7%	13.3%	0.0%	0.0%
ERRFI1	12.5%	0.0%	0.0%	0.0%	81.3%	6.3%	0.0%	0.0%
FLT3	12.0%	4.0%	0.0%	0.0%	76.0%	4.0%	4.0%	0.0%
KIT	0.0%	2.8%	0.0%	0.0%	88.9%	2.8%	5.6%	0.0%
MET	4.2%	4.2%	0.0%	0.0%	75.0%	16.7%	0.0%	0.0%
PTPN11	0.0%	8.3%	0.0%	0.0%	91.7%	0.0%	0.0%	0.0%
RET	2.9%	0.0%	0.0%	0.0%	94.1%	2.9%	0.0%	0.0%
RIT1	0.0%	0.0%	0.0%	0.0%	80.0%	10.0%	10.0%	0.0%
ROS1	5.1%	1.7%	0.0%	0.0%	88.1%	3.4%	1.7%	0.0%

	Late-Onset NHW Samples								
	Frame Shift Deletion	Frame Shift Insertion	In Frame Del	In Frame Ins	Missense Mutation	Nonsense Mutation	Nonstop Mutation	Splice Region	Splice Site
IGF1R	8.8%	7.1%	0.0%	0.0%	80.5%	2.7%	0.0%	0.0%	0.9%
ERBB2	3.7%	4.4%	0.0%	0.0%	91.1%	0.0%	0.0%	0.0%	0.7%
EGFR	1.1%	0.0%	2.3%	1.1%	83.0%	6.8%	0.0%	1.1%	4.5%
ERBB3	4.6%	1.3%	2.6%	0.0%	87.4%	0.7%	0.0%	0.0%	3.3%
ERBB4	6.5%	2.4%	0.0%	0.0%	80.0%	6.5%	0.0%	0.0%	4.7%
BRAF	2.4%	2.1%	0.0%	0.0%	94.6%	0.3%	0.0%	0.0%	0.6%
FGFR1	3.6%	0.0%	0.0%	0.0%	92.7%	0.0%	0.0%	0.0%	3.6%
FGFR2	4.2%	2.1%	0.0%	0.0%	85.4%	4.2%	0.0%	0.0%	4.2%
FGFR3	5.8%	7.2%	1.4%	0.0%	82.6%	0.0%	0.0%	1.4%	1.4%
FGFR4	9.6%	2.7%	0.0%	0.0%	83.6%	2.7%	0.0%	0.0%	1.4%
HRAS	0.0%	0.0%	0.0%	0.0%	95.0%	5.0%	0.0%	0.0%	0.0%
KRAS	0.0%	0.2%	0.2%	0.5%	99.1%	0.0%	0.0%	0.0%	0.0%
MAP2K1	10.8%	0.0%	13.5%	0.0%	73.0%	0.0%	0.0%	0.0%	2.7%
MAP2K2	6.7%	0.0%	0.0%	0.0%	90.0%	0.0%	0.0%	0.0%	3.3%
MAPK1	0.0%	0.0%	9.1%	0.0%	81.8%	9.1%	0.0%	0.0%	0.0%
MAPK3	5.3%	0.0%	10.5%	0.0%	78.9%	5.3%	0.0%	0.0%	0.0%
NF1	8.8%	6.2%	0.0%	0.5%	64.2%	13.5%	0.0%	0.0%	6.7%
NRAS	0.0%	0.0%	0.0%	0.0%	100.0%	0.0%	0.0%	0.0%	0.0%
SHC1	87.5%	0.0%	0.0%	0.0%	12.5%	0.0%	0.0%	0.0%	0.0%
GRB2	0.0%	0.0%	0.0%	0.0%	100.0%	0.0%	0.0%	0.0%	0.0%
NTRK1	0.0%	0.0%	0.0%	0.0%	90.5%	7.9%	0.0%	0.0%	1.6%
NTRK2	0.0%	0.0%	0.0%	0.0%	96.6%	3.4%	0.0%	0.0%	0.0%
PDGFRA	1.1%	2.3%	0.0%	0.0%	81.8%	10.2%	0.0%	0.0%	4.5%
RAC1	10.0%	0.0%	0.0%	0.0%	90.0%	0.0%	0.0%	0.0%	0.0%
RAF1	3.9%	3.9%	0.0%	0.0%	84.3%	3.9%	0.0%	0.0%	3.9%

RASA1	18.6%	4.9%	1.0%	0.0%	61.8%	10.8%	0.0%	0.0%	2.9%
SOS1	22.8%	5.3%	0.0%	0.0%	66.7%	5.3%	0.0%	0.0%	0.0%
ALK	10.3%	4.4%	0.0%	0.0%	78.7%	2.9%	0.0%	2.2%	1.5%
ARAF	34.4%	3.1%	0.0%	0.0%	56.3%	1.6%	0.0%	0.0%	4.7%
CBL	6.5%	4.3%	0.0%	6.5%	71.7%	10.9%	0.0%	0.0%	0.0%
ERRFI1	24.3%	2.7%	0.0%	0.0%	73.0%	0.0%	0.0%	0.0%	0.0%
FLT3	11.8%	0.0%	0.0%	0.0%	82.4%	2.0%	0.0%	0.0%	3.9%
KIT	0.0%	5.2%	0.0%	0.0%	85.7%	6.5%	0.0%	0.0%	2.6%
MET	10.0%	1.4%	1.4%	2.9%	72.9%	11.4%	0.0%	0.0%	0.0%
PTPN11	2.6%	0.0%	0.0%	0.0%	89.7%	5.1%	0.0%	2.6%	0.0%
RET	0.0%	0.0%	1.1%	0.0%	93.6%	4.3%	0.0%	0.0%	1.1%
RIT1	0.0%	6.3%	6.3%	0.0%	62.5%	12.5%	0.0%	6.3%	6.3%
ROS1	9.7%	2.8%	0.7%	0.0%	75.0%	9.0%	0.7%	0.0%	2.1%

Table S4. Microsatellite instability (MSI) status and MSI scores in early-onset and late-onset colorectal cancer among Hispanic/Latino (H/L) and non-Hispanic White (NHW) patients. This table summarizes MSI classification (stable, instable, indeterminate, not reported, and unavailable) and quantitative MSI scores (average, median, minimum, maximum) across four patient subgroups: early-onset H/L, late-onset H/L, early-onset NHW, and late-onset NHW.

	Early-Onset HL	Late-Onset HL	Early-Onset NHW	Late-Onset NHW
**MSI Type**				
Stable	97 (70.3%)	126 (76.8%)	752 (83.8%)	1608 (72.7%)
Instable	11 (8.0%)	23 (14.0%)	74 (8.2%)	282 (12.7%)
Indeterminate	8 (5.8%)	2 (1.2%)	14 (1.6%)	58 (2.6%)
Do not report	0 (0.0%)	0 (0.0%)	10 (1.1%)	10 (0.5%)
Unavailable	22 (15.9%)	13 (7.9%)	47 (5.2%)	255 (11.5%)
**MSI Score**				
Average	3.80	4.65	2.98	4.85
Median	0.62	0.455	0.33	0.51
Minimum	0	0	0	0
Maximum	43.03	48.4	48.01	53.44
